# Humoral and cell mediated immune response to SARS-CoV-2 vaccination in patients with immune-mediated diseases

**DOI:** 10.3389/fimmu.2026.1813924

**Published:** 2026-05-18

**Authors:** Dorey A. Glenn, Yichun Hu, Meghan E. Free, Lakshmanane Premkumar, Tara M. Narowski, Grayson M. Coleman, Sandra Elmore, Mary M. Collie, Donna A. Culton, Nicole M. Orzechowski, Eveline Y. Wu, Ronald J. Falk, Donna O. Bunch, Vimal K. Derebail

**Affiliations:** University of North Carolina at Chapel Hill, Chapel Hill, NC, United States

**Keywords:** autoimmune, cellular immunity, humoral immunity, SARS-CoV-2, vaccine, vasculitis

## Abstract

**Background:**

Vaccination against SARS-CoV-2 induces an immune response that is protective against severe disease in healthy populations. However, humoral and cellular immune responses in individuals with immune-mediated diseases receiving immunosuppressive medications are not well understood.

**Methods:**

We conducted a single-center, prospective observational cohort study of pediatric and adult patients with vasculitis, glomerular disease, or other immune-mediated diseases. Antibody response assessed by viral neutralization and, in a subset, cellular immunity to SARS-CoV-2 vaccination were assessed before and at time points following initial and booster vaccination.

**Results:**

Between March 2021 and June 2022, 80 individuals with immune-mediated diseases and 12 healthy controls were enrolled and followed for a median of 11.97 months (IQR 7.64, 14.05). Following vaccination, the median percent angiotensin-converting enzyme 2 (ACE2) neutralization at V1 (1–3 months post vaccination) for patients in the immune-mediated disease cohort and healthy controls were 46.9% (IQR 0.65, 95.6) and 95.9% (IQR 94.6, 96.5), respectively. Of 26 individuals with anti-CD20 therapy exposure or laboratory evidence of B cell depletion at time of vaccination, only 11.9% had protective neutralization titers at V1. After adjustment for age, sex, BMI, race, vaccine type, and number of comorbidities, anti-CD20 exposure at time of initial vaccination remained significantly associated with a lower odds of ACE2 neutralization ≥30% at V1. The median T-ELISpot counts (RBD) at V1 for patients with immune-mediated diseases and healthy controls were comparable (16 [IQR 12, 37] and 16 [IQR 4.5, 23], respectively).

**Conclusions:**

Vaccination against SARS-CoV-2 during treatment with anti-CD20 antibody therapy was associated with impaired humoral immunity, but T cell responses were qualitatively preserved despite immunosuppressant exposure.

## Introduction

Vaccination against SARS-CoV-2 elicits high levels of protective antibodies and robust T cell responses in healthy individuals and is highly effective at reducing severe disease in the general population (1). Patients with immune-mediated diseases were generally excluded from initial phase 2/3 efficacy studies of SARS-CoV-2 vaccines and thus effectiveness in this population is not well understood (2). Vaccine effectiveness studies in immunosuppressed patients have overwhelmingly focused on humoral immunity, measuring total antibody formation and functional antibody responses against the spike protein. Extended primary series strategies have demonstrated both enhanced humoral responses and *de novo* seroconversion in immunocompromised patients without a detectable humoral response after a standard primary series (3).

Humoral immune responses to SARS-CoV-2 vaccination are subject to effects of T and B cell-directed immunosuppressive medications (4, 5). Cell-mediated immunity is critical for protection against hospitalization or death from SARS-CoV-2 infection (1), but assays require patient-derived cells which are technically challenging to biobank, and laborious to perform. While T cell responses have not been incorporated into approval pathways for vaccine development and licensure, they may inform vaccine-induced protection among patients exposed to immunosuppressive or immunomodulatory medications when humoral immunity is impaired (6).

We conducted a single-center observational cohort study to characterize the humoral and cell-mediated immune responses to SARS-CoV-2 vaccines (Moderna [mRNA-1273], Pfizer-BioNTech [BNT162b2] and Janssen [Ad26.COV2.S]) in patients with vasculitis, glomerular disease, and other immune-mediated diseases. Our objectives were to identify sociodemographic and clinical risk factors for lower rates of seroprotection and loss of vaccine-induced immunity. We hypothesized that among patients with immune-mediated disease, immunosuppressive therapies, particularly anti-CD20 directed therapies, will be associated with impaired humoral and altered cellular immune responses compared to healthy controls.

## Methods

### Study design

We conducted a single-center, prospective observational cohort study of pediatric and adult patients with vasculitis, glomerular disease, or other immune-mediated diseases. Participants were enrolled before or at the time of SARS-CoV-2 vaccination. Antibody response and, in a subset, cellular immunity to SARS-CoV-2 vaccination was assessed before and at time points following initial and booster vaccination.

When SARS-CoV-2 vaccination was expected to occur at a date later than the enrollment visit, a separate V0 visit was conducted to obtain baseline biosamples. Otherwise, enrollment and V0 were conducted contemporaneously. Study visits and biospecimens were collected at V1 (1–3 months), V2 (6–9 months), and V3 (12–15 months) following initial vaccination. Participants who received a third mRNA vaccine dose per CDC guidelines had their visit schedule “reset” to the booster visit (BV) schedule. We attempted to collect a biospecimen within 8 weeks of their booster vaccination (BV0). Study visits and biospecimens were then collected at BV1 (1–3 months), BV2 (4–6 months), and BV3 (7–9 months) following booster vaccination.

### Study population

Pediatric (≥ 2 to <18 years) and adult patients (≥18 years) with glomerular disease, vasculitis, and other immune-mediated diseases were eligible for enrollment. Vasculitis patients could have small-, medium-, or large-vessel vasculitis. Patients with glomerular diseases included those with a diagnosis of IgA nephropathy, focal segmental glomerulosclerosis, minimal change disease, membranous nephropathy, or idiopathic nephrotic syndrome. Additional eligible immune-mediated conditions included systemic lupus erythematosus, rheumatoid arthritis, Sjögren’s syndrome, scleroderma, mixed connective tissue disorder and overlap autoimmune disorders. Individuals with a history of splenectomy, cancer with exposure to chemotherapy in the prior two years, active coronavirus disease 2019 (COVID-19) infection at enrollment, uncontrolled HBV, HCV, or HIV infection, solid organ or bone marrow transplant, or those receiving renal replacement therapy were excluded. Patients were also excluded if they had received blood, blood products, or intravenous immunoglobulin in the prior three months or an investigational immunomodulatory drug in the prior 12 months.

Healthy volunteers were recruited within an expected age and sex distribution to study participants. Volunteers were considered eligible if they did not have a health history that would be anticipated to influence response to vaccination in the opinion of the investigators.

### Immunosuppression exposure

Immunosuppressive medication exposure was captured by patient report at each study visit and medication start/stop dates abstracted from the electronic medical record. Immunosuppression exposure at the time of SARS-CoV-2 vaccination was defined using the time-period from 14 days prior to the first SARS-CoV-2 vaccination to the date of the V1 visit. When concurrent immunosuppressants were identified, a hierarchical algorithm was applied to prioritize immunosuppressant exposure (rituximab > mycophenolate (MMF) >azathioprine >methotrexate>other). Other immunosuppressive medications included budesonide, infliximab, ixekizumab, prednisone, and tacrolimus. Rituximab exposure was defined from date of infusion up to 6 months thereafter, or evidence of B-cell depletion (CD19 < 1% or absolute CD19 cell count < 10 cells/μL +/- 30 days) from first SARS-CoV-2 vaccination.

### Covariates

Participant demographics (age, sex, race, ethnicity), vaccination history, and medical history, including comorbidities and a COVID-19 illness questionnaire, were obtained at enrollment. Disease onset, prior disease treatment, and past hospitalizations related to infection were obtained from patient report and abstraction from the electronic medical record. Updated medical history, including interim vaccinations, infections and medication changes were obtained at follow-up visits (V1–3 and BV0-3).

### Outcome: humoral and cell-mediated immunity

Binding and neutralizing antibody titers were measured prior to vaccination and at three standardized timepoints following completion of the standard vaccination course (1–3 month, 6–9 months, and 12–15 months). For those receiving a 3^rd^ mRNA booster vaccine dose, an attempt was made to obtain a biosample within 8 weeks before the booster vaccination and after 1-3, 4–6 and 7–9 months. Seroconversion was defined as a ≥4-fold change in antibody titer from baseline (pre-vaccination titer) to 1–3 months after completing the vaccination series.

### Antibody assays

SARS-CoV-2 spike antibodies targeting the receptor-binding domain (RBD) and the N-terminal domain (NTD) were tested in ELISA as previously described (7). Neutralizing antibodies to SARS-CoV-2 were measured semi-quantitatively using a Surrogate Virus Neutralization Test (sVNT) c-Pass kit (GenScript) (8). The assay detects serum antibodies that block the interaction of the RBD on the spike protein with the human angiotensin-converting enzyme 2 (ACE2) receptor. A signal inhibition cutoff value of 30% was manufacturer-defined, and previously validated in multiple cohorts (9).

### Cellular assays

#### T-ELISpot assay

To assess cell-mediated responses to SARS-CoV-2 vaccination, we quantified T-cell activation as measured by IFN-γ release following exposure to RBD peptide using a *T-ELISpot assay.* Peripheral blood mononuclear cells (PBMCs) were isolated from CPT Tubes (BD) and cryopreserved in 10% DMSO + FBS at a cell concentration of 5E6/mL. Frozen PBMCs were thawed and washed at the start of each assay with a cell media containing RPMI 1640 supplemented with 10% fetal bovine serum and HEPES buffer solution. ELISpot assays were conducted using MabTech ELISpot Pro for Human IFNy assay (MabTech, 3420-2HPT-10). Prior to plating, the ELISpot plate was washed with PBS and blocked with cell media. Samples at a cell concentration of 3.5x10^5^/100μL in cell media were added in a descending vertical order to the plate as follows: a negative control mock well containing 100μL of cell media, a positive control well containing 100μL of mAB CD3–2 master mix, 80μL of the CEFX PepMix (JPT peptides), or 80μL of the RBD PepMix (JPT peptides). After adding these conditions to their respective wells, 100μL of cells were added. Plates were then sealed in parafilm and incubated for 18 hours at 37°C. Following the 18-hour incubation, plates were washed with PBS and then developed with the detection antibody master mix (7-B6-ALP diluted 1:200). Plates rested at room temperature for two hours and then were washed with PBS and received 100μL of substrate per well and developed over ten minutes. After extensive washing under distilled water, the plates were stored away from light to dry before being imaged.

### Flow cytometry studies

A flow cytometry panel containing antibodies corresponding to eight cytokines along with CD4 and CD8 to identify helper and cytotoxic T cells, respectively, was used. T cell subsets of interest included Th1 (identified by CD4^+^ secreting IFN-γ, IL-2, and TNF-α), Th17 (identified by CD4^+^ secreting IL-22, IL-17F, and IL-17A), and Tfh (identified by CD4^+^ secreting IL-21 and IL-10). CD8^+^ secreting IFN-γ, IL-2, and TNF-α were also measured. Two FMOs (fluorescence minus one) were used under mock and PMA/ionomycin conditions. The following controls and stimulants were used: a negative control mock well containing cell media, a positive control well containing PMA/ionomycin (2µL/mL), a CEFX well (1µL/250µL), and a RBD well (1µL/250µL).

Once thawed, cells were resuspended to a concentration of 5x10^6^/mL of cell media and were incubated under the same conditions as the ELISpot analyses. The cells were resuspended to the same concentration (5x10^6^/mL of cell media) and left to incubate for one hour. Afterwards, GolgiPlug (BD Biosciences, 555029) was added for an additional three hours at a concentration of 1µL of GolgiPlug to 1 mL of cell suspension.

For flow cytometry staining, cells were blocked with FcX TruStain (BioLegend) prior to surface staining. After blocking, master mixes containing the appropriate surface antibodies were applied and incubated. After a 30-minute incubation on ice, cells were washed with FACS buffer. Wells were resuspended in 100µL BD Perm/Fix solution and incubated protected from light for 30 minutes. Cells were then washed with Perm/Wash prior to intracellular staining. Anti-cytokine antibodies were added for intracellular staining on ice for 30 minutes. After final washes, cells were acquired on a Thermo Fisher Attune NxT and analyzed by FlowJo.

### Statistical methods

Demographic and clinical characteristics at study enrollment were summarized using median and interquartile range (IQR) for continuous variables and frequency and percentage for categorical variables. Median fold-change in neutralization titers were calculated for V1 and V2 timepoints relative to baseline (V0). Missing baseline values were imputed using the median value from all non-missing baseline participants. All 0% neutralization results at V0 were set to 1 for the purpose of determining fold change. Statistical tests and visual charts were used for group comparisons. The Wilcoxon rank-sum or Kruskal-Wallis tests were performed depending on the number of groups (two or three groups). A multivariable logistic regression model adjusted for demographic and clinical characteristics was developed to identify factors for suboptimal % ACE2 neutralization at V1, defined as a < 30% binding inhibition (primary analysis). A secondary analysis of longitudinal % ACE2 neutralization was performed using a linear mixed-effects model. Time was defined as months from initial SARS-CoV-2 vaccination to the date of measured neutralization over 12 months of study follow-up, with pre-vaccination values assigned a time value of 0. Immunosuppression exposure at the time of vaccination was modeled as a fixed effect with interactions included for linear and quadratic time terms. The model was additionally adjusted for age and sex. Subject‐specific heterogeneity was modeled with random intercepts and random slopes for time for each participant. Fixed effects were tested using Kenward–Roger adjusted denominator degrees of freedom to account for small-sample bias (10). Parameters were estimated by restricted maximum likelihood using SAS PROC MIXED.

To define low responses for T-ELISpot and flow cytometry assays for patients with immune-mediated disease, threshold values were set at the lower IQR of values from the healthy control group. SAS version 9.4 (SAS Institute, Cary, NC) was used for all statistical analyses. Figures were created using SAS and GraphPad Prism version 10.0.0, (GraphPad Software, Boston, Massachusetts).

## Results

Between March 2021 and June 2022, 83 individuals with immune-mediated disease were enrolled. Three individuals with no follow-up data were excluded, leaving 80 individuals with immune-mediated disease and 12 healthy controls included in the analysis. Median follow-up was 11.97 months (IQR 7.64, 14.05) for the full cohort. (**Table 1**) The median age at study enrollment was 62 years (IQR 38, 71) and 53 years (IQR 35, 64) for patients with immune-mediated conditions and healthy control subjects, respectively. Of those with immune-mediated conditions, 71% were female, 14% were Black, and 8% were Hispanic. Diagnoses were categorized into five subgroups, including vasculitis (54%), glomerular disease (18%), rheumatologic disease (15%), dermatological disease (11%), and gastrointestinal disease (3%). The most frequent comorbidities among those with immune-mediated conditions were hypertension (49%) and a history of malignancy (9%). All but one participant received an mRNA-based SARS-CoV-2 vaccine as their primary series vaccination. Booster vaccination was recorded in 69 of 80 participants in the immune-mediated disease cohort, and 8 of 12 healthy controls. The median time from first to second SARS-CoV-2 vaccination in the immune-mediated disease and healthy control groups were 0.82 months (IQR 0.69, 0.92) and 0.74 months (IQR 0.69, 0.84), respectively. The median time from second vaccination to first booster vaccination was 6.2 months (IQR 5.2, 6.9) for the immune-mediated disease group and 10.1 months (IQR 9.5, 10.8) for the healthy control group.

**Table 1:** Cohort characteristics.

Characteristic	Immune-Mediated Disease Cohort	Healthy Controls
	(N = 80)	(N = 12)
Age, years	62.4 (37.5, 70.6)	52.8 (34.5, 63.7)
BMI	28.7 (24.1, 33.9)	24.5 (23.0, 26.3)
Female Sex, n (%)	57(71.3%)	8 (66.7%)
Race, n (%)		
White	64(80.0%)	7 (58.3%)
Black	11(13.8%)	2 (16.7%)
Multiracial/Other	5(6.3%)	3 (25.0%)
Ethnicity, n (%)		
Hispanic/Latino	6(7.5%)	3 (25.0%)
Disease Type, n (%)*		
Dermatological Disease	9(11.3%)	0 (0.0%)
Gastrointestinal Disease	2(2.5%)	0 (0.0%)
Glomerular Disease	14(17.5%)	0 (0.0%)
Rheumatologic Disease	12(15.0%)	0 (0.0%)
Vasculitis	43(53.8%)	0 (0.0%)
Comorbidities, n (%)		
Hypertension	39(48.8%)	0 (0.0%)
DM	3(3.8%)	0 (0.0%)
Hepatitis A/B/C	1(1.3%)	0 (0.0%)
Cancer	7(8.8%)	0 (0.0%)
Tobacco Exposure	3(3.8%)	0 (0.0%)
Other	5(6.3%)	0 (0.0%)
Vaccine Type, n (%)		
Pfizer	48(60.0%)	11 (91.7%)
Moderna	31(38.8%)	1 (8.3%)
Johnson & Johnson	1(1.3%)	0 (0.0%)
Coronavirus Infection Prior to Enrollment, n(%)	1(1.3%)	0 (0.0%)
Disease status, n (%)		
Active Disease	21(26.3%)	NA
Disease Remission	54(67.5%)	NA
Missing/Not Applicable	5(6.3%)	NA
Immunosuppression at Time of Vaccination, n(%)		
Rituximab**	24(30.0%)	NA
Mycophenolate	22(27.5%)	NA
No Immunosuppression	23(28.8%)	NA
Other Immunosuppression	11(13.8%)	NA

*Included the following diseases: Granulomatosis with polyangiitis (GPA), Microscopic polyangiitis (MPA), Eosinophilic granulomatosis with polyangiitis (EGPA), Lupus Nephritis, Systemic lupus erythematosus (SLE), IgA nephropathy, Minimal Change Disease, Membranous Nephropathy, FSGS, MPGN, C3GN, Glomerular endotheliosis, Rheumatoid Arthritis, Ankylosing Spondylitis, Psoriatic arthritis, Pemphigus vulgaris, Mucous membrane pemphigoid, Systemic sclerosis, Sarcoidosis, Large vessel vasculitis, Giant cell arteritis (GCA), Type 2 Cryoglobulinemic vasculitis, Autoimmune cytopenia, Autoimmune enteropathy, Crohn’s disease. ** Two additional patients demonstrated laboratory evidence of B cell depletion at the time of vaccination.

Across all study follow-up, 96.4% of the immune-mediated disease cohort had received some form of immunosuppressive exposure. Immunosuppression at the time of vaccination was identified in 57 of 80 individuals (71%) with immune-mediated conditions and included rituximab (30%), MMF (23%), azathioprine (5%), tacrolimus (1.3%), prednisone (4%), methotrexate (5%**),** and other immunosuppressive medication exposures (4%). Approximately one third (29%) of those in the immune-mediated disease cohort were not receiving an immunosuppressive medication at the time of vaccination.

### Humoral immune response

**Figure 1:**
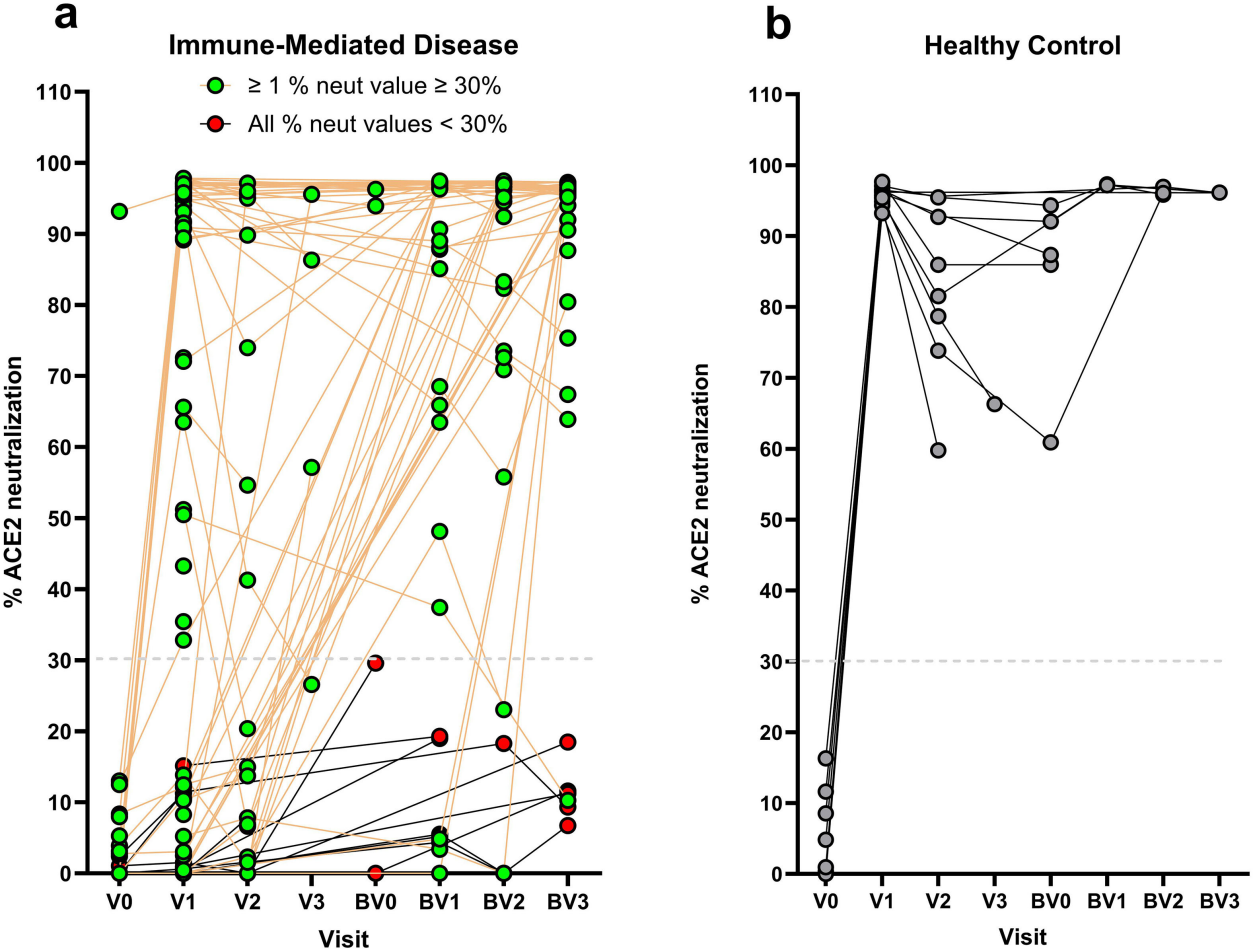
**(a, b)** Percent neutralizing anti-spike antibody across study follow up in **(a)** individuals with immune-mediated disease, and **(b)** healthy controls. In **(a)** lines connect percent neutralizing values for individual patients. Green circles represent patients with at least one measure above the seroprotective threshold (i.e. 30%). Red circles represent patients with all measures below the seroprotective threshold.

**Figure 2:**
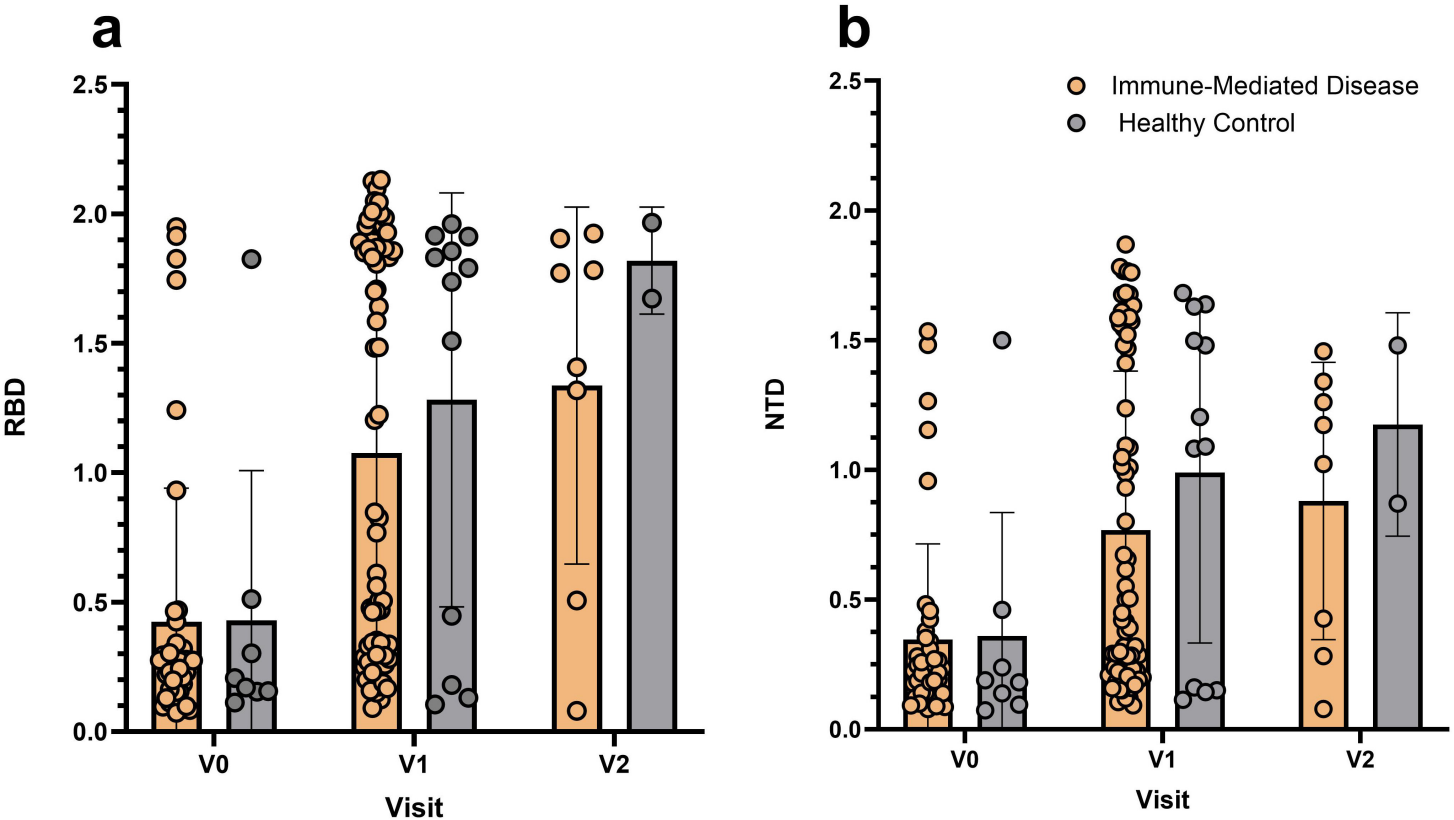
**(a, b)** Optical density measurements of **(a)** receptor-binding domain (RBD) and **(b)** N-terminal domain (NTD).

Percent ACE2 neutralization activity by study visit is shown in **Figure 1**. The proportion of missing baseline neutralization activity values at V0 (i.e. pre-vaccination) was 64% and 42% for the immune-mediated disease cohort and healthy control group, respectively. The median percent ACE2 neutralization at V1 for patients with immune-mediated disease and healthy controls were 46.9% (IQR 0.65, 95.6) and 95.9% (IQR 94.6, 96.5), respectively. Summary measures by study visit are shown in **Supplementary Table 1** and depicted in **Supplementary Figure 1**. The median fold-change in percent ACE2 neutralization activity from V0 to V1 was 12.5 (IQR 0.7, 94.9) for the immune-mediated disease cohort and 19.8 (IQR 11.3, 20.1) for healthy controls. The median fold-change from V0 to V2 was 7.8 (IQR 0, 20.4) for the immune-mediated disease cohort and 19.7 (IQR 6.4, 91.4) for healthy controls (**Table 2**). The median fold-change in optical densities (OD) of RBD and NTD at V1 and V2 relative to V0 are summarized in **Table 2**. RBD and NTD values across all study visits is summarized in **Supplementary Table 2** and depicted in **Figures 2a, b**.

**Table 2:** Median fold change in % ACE2 neutralization, and RBD/NTD optical density measures at Visit V1 and V2 relative to pre-vaccination visit V0.

Parameter	Measure	Fold change compared to V0 median (IQR)					
		V1			V2		
		Immune-Mediated Disease Cohort	Healthy Control	p	Immune-Mediated Disease Cohort	Healthy Control	p
% ACE2 Neutralization	n	77	11		26	7	
	Median (IQR)	12.5 (0.7, 94.9)	19.8 (11.3, 20.1)	0.4108	7.8 (0, 20.4)	19.7 (6.4, 91.4)	0.1634
RBD	n	31	8		5	1	
	Median (IQR)	5.0 (1.3, 7.0)	2.6 (1.0, 11.7)	1.000	6.7 (4.6, 11.4)	6.5 (6.5, 6.5)	1.000
NTD	n	31	8		5	1	
	Median (IQR)	2.5 (1.0, 5.3)	2.7 (0.7, 10.0)	0.7942	5.4 (1, 10.9)	20 (20.0, 20.0)	0.2416

RBD, receptor-binding domain; NTD, N-terminal domain. *Missing values for baseline neutralization activity (V0) (n=59/95). Baseline values of 0, set to 1 for purposes of calculating fold change. Missing values at V0 were imputed using the median neutralizing values at V0.

**Figure 3:**
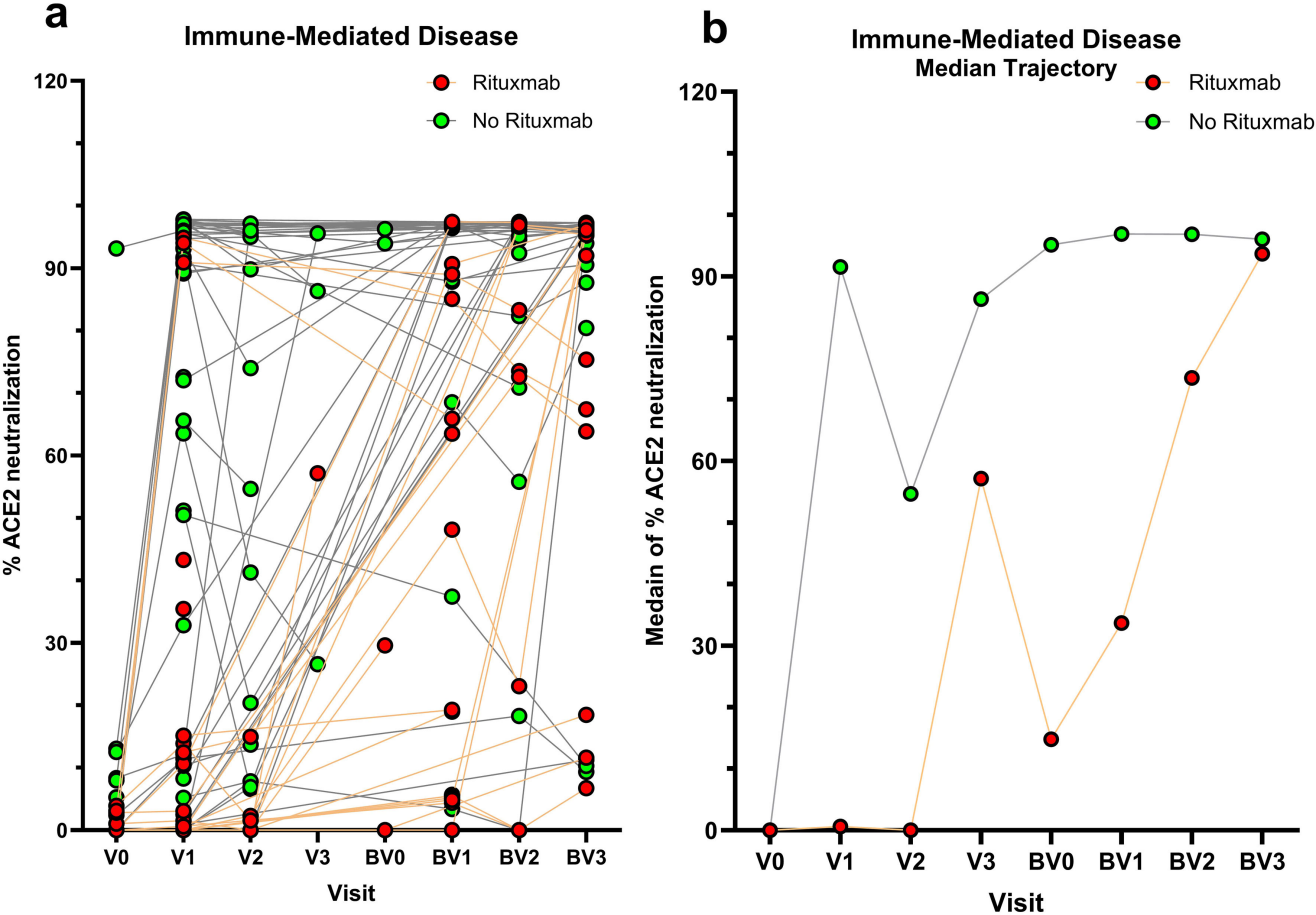
**(a)** Percent ACE2 neutralization and **(b)** median percent ACE2 neutralization across study follow-up among individuals with immune-mediated disease comparing those with vs. without anti-CD20 therapy exposure at time of vaccination.

Of 26 individuals with anti-CD20 therapy exposure or laboratory evidence of B cell depletion at time of vaccination, 11.9% had protective neutralization titers at V1. Demographic and clinical characteristics of patients with rituximab exposure at time of vaccination stratified by percent ACE2 neutralization at V1 are summarized in **Supplementary Table 3** and a comparison of percent ACE2 neutralization in those with versus without rituximab exposure across follow-up is shown in **Figures 3a, b**. Median time from the most proximate rituximab infusion to first SARS-CoV-2 vaccination was shorter in those with % neutralization at V1 < 30% compared to ≥ 30% [2.94 months (IQR 2.11, 4.62) vs. 4.13 months (IQR 2.63, 6.45)]. In univariate analysis, rituximab exposure at time of vaccination was significantly associated with a lower odds of achieving a percent ACE2 neutralization ≥30% at V1 (OR 0.13 (95% CI 0.03 - 0.53). In the primary analysis, after adjustment for age, sex, BMI, race, vaccine type, and number of comorbidities, rituximab exposure at time of initial vaccination remained significantly associated with a lower odds of achieving a percent ACE2 neutralization ≥30% at V1. (**Supplementary Table 4**).

In a secondary analysis of longitudinal post-vaccination response trajectories, % ACE2 neutralization trajectories differed significantly by immunosuppression exposure. Patients not receiving immunosuppression at the time of vaccination demonstrated an initially increased % ACE2 neutralization (month: β=23.2, p=0.0015) with subsequent downward curvature (Month²: β=−1.8, p=0.037), indicating a nonlinear trajectory that rose initially and then slowed. Baseline % ACE2 neutralization did not differ significantly by immunosuppression category. However, time trends differed by immunosuppression exposure group. Those individuals exposed to rituximab at the time of vaccination exhibited an attenuated early increase (month×rituximab: β=−23.1, p=0.0047) and significantly different curvature (Month²×rituximab: β=1.9, p=0.040), compared to those without immunosuppression exposure, consistent with a blunted early vaccine response. The MMF/azathioprine exposure group demonstrated a non-significant trend toward reduced neutralization (Month × MMF/azathioprine: β = −16.2, SE = 8.5, p = 0.059). Age and sex were not significantly associated with % ACE2 neutralization after adjustment (age: p = 0.38; sex: p = 0.26). (**Supplementary Table 5** and **Supplementary Figure 2**).

Of 22 individuals with MMF exposure at time of vaccination, 63.6% had protective neutralization activity at some point during follow-up compared to 100% of healthy controls.

Of 80 patients in the immune-mediated cohort, 69 received a booster vaccination. Of those, 50 had at least 1 measure of ACE2 neutralization following booster vaccination. In those with a neutralizing response <30% at all three post-vaccination timepoints (V1, V2, and V3), 20 of 32 (62%) developed what was considered to be an adequate response (≥30%) at any post-booster timepoint (BV1, BV2, or BV3). In those patients with an adequate response (≥30%) at one or more post-vaccination timepoints (V1, V2, or V3), 7 of 37 (19%) failed to achieve seroprotection (≥30%) at any post-booster timepoint (BV1, BV2, or BV3), despite receiving a booster dose.

### Cell-mediated response

#### T-ELISpot assay

**Figure 4:**
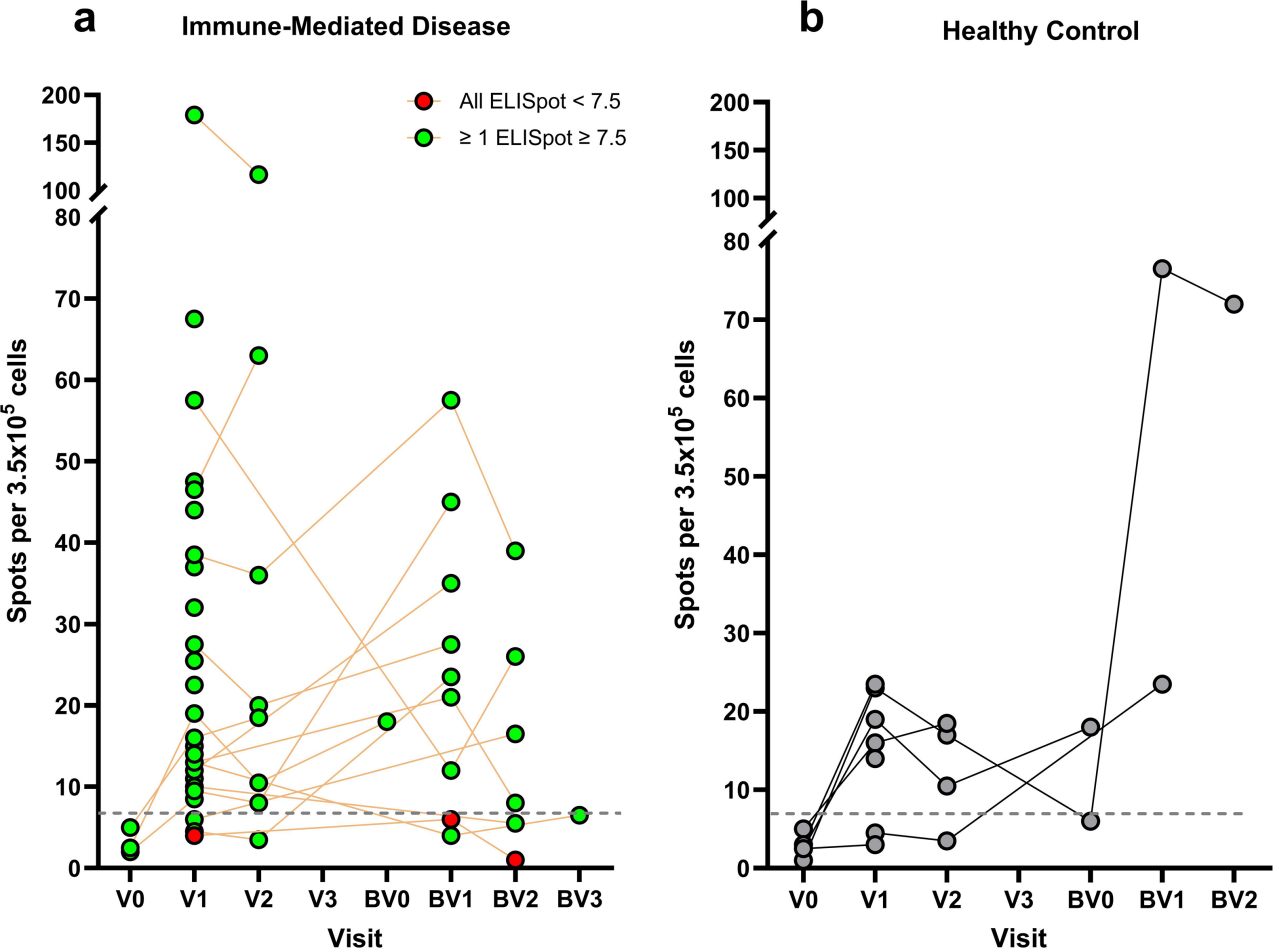
T-ELISpot measurements across study follow up in **(a)** individuals with immune-mediated disease, and **(b)** healthy controls. In **(a)** lines connect results for individual patients. Green circles represent patients with at least one measurement above the defined seroprotective threshold (i.e. 7.5). Red circles represent patients with all values below the seroprotective threshold.

T-ELISpot measurements of IFN-γ secretion across study follow up in individuals with immune-mediated disease and healthy controls are shown in **Figures 4a, b**. The median T-ELISpot counts (RBD) at V1 for patients with immune-mediated disease and healthy controls were 16 (IQR 12, 37) and 16 (IQR 4.5, 23), respectively. Summary measures by study visit are summarized in **Supplementary Table 6**. A comparison of T-ELISpot measures from 23 patients with immune-mediated disease with both T-ELISpot and ACE2 neutralization assays at V1 is presented in **Table 3**. There was a trend towards higher T-ELISpot counts in those with a percent ACE2 neutralization ≥80%, though not statistically significant.

**Table 3:** Comparison of T-ELISpot results for 23 patients with immune-mediated disease with biosamples at V1, stratified by %ACE2 neutralization.

	Group	Number (N)	T-ELISpot Value at V1	p
			Median (IQR)	0.1004
% ACE2 Neutralization at V1	<30%	10	12.5(9.5, 38.5)	
	30-80%	3	12.0(6.0, 13.0)	
	≥80%	10	32.0(15.0, 46.5)	

ACE2, angiotensin-converting enzyme 2.

### Flow cytometry studies

**Figure 5:**
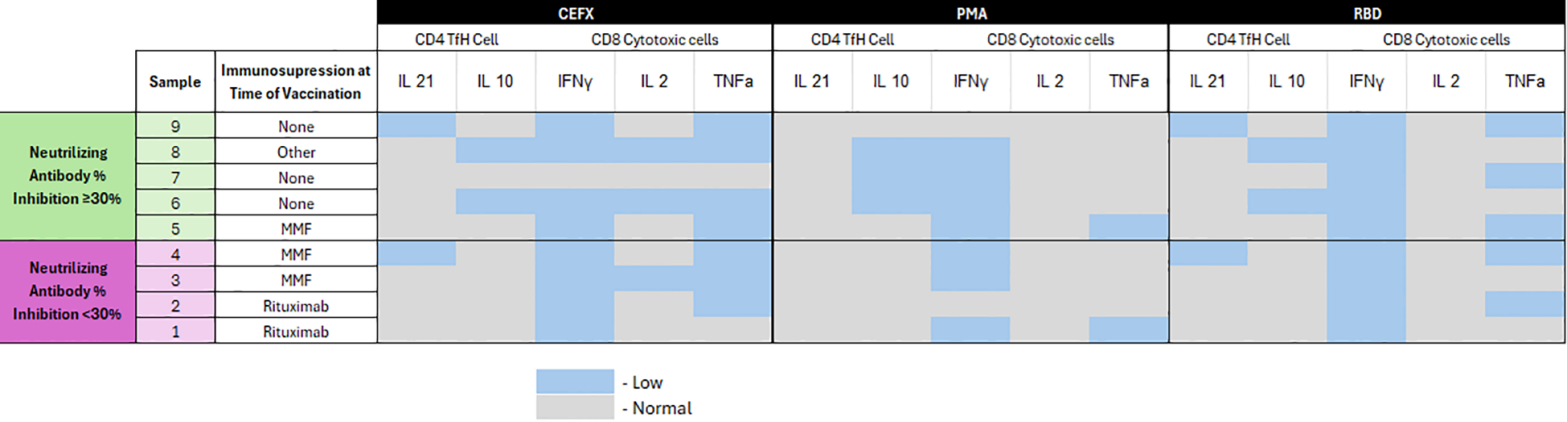
Heat map of flow cytometry analysis of T cells following SARS-CoV-2 vaccination stratified by anti-spike neutralizing antibody response. Low responses were defined as values below the lower IQR of values from the healthy control group. SAS v9.4 (SAS Institute, Cary, NC) was used for all statistical analyses.

T cell responses to control (CEFX, PMA) and spike protein antigen (RBD) were analyzed from nine participants with immune-mediated disease and three healthy controls. Four of nine participants with immune-mediated disease had demonstrated sub-optimal neutralizing antibody titers. Visual comparison of T cell responses within CD4 (IL-21, IL-10) and CD8 (IFN-γ, IL-2, TNF-α) are presented in **Figure 5**. Cellular responses were comparable in those who did and did not demonstrate a neutralizing antibody response. Low IFN-γ responses across PMA/ionomycin, CEFX, and RBD conditions suggests broadly impaired immune fitness among patients in the immune-mediated cohort compared to controls.

### Coronavirus infections

Of 80 participants with immune-mediated disease, 1 individual reported a SARS-CoV-2 infection prior to enrollment and demonstrated a protective percent neutralizing response (i.e. ≥30%) at V0. Self-reported coronavirus infections occurring at any point after initial vaccination were reported in 13 (37.1%) and 4 (9.5%) of those with % neutralization < 30% compared to ≥ 30% at V1, respectively. (**Table 4**) Of 12 healthy control subjects, 1 self-reported coronavirus infection was recorded following booster vaccination.

**Table 4:** Self-reported SARS-CoV-2 infection prior to and following SARS-CoV-2 vaccination among the immune-mediated disease cohort stratified by percent ACE2 neutralization at V1.

SARS-CoV-2 infection history	% ACE2 neutralization at V1	
	<30%	≥30%
	(N = 35)	(N = 42)
Coronavirus Infection prior to Study Enrollment*		
No	34 (97.14%)	38 (90.48%)
Yes, definitely	1 (2.86%)	3 (7.14%)
Yes, probably	0 (0.00%)	1 (2.38%)
Coronavirus Infection Following Initial Vaccination**		
Yes	13 (37.14%)	4 (9.52%)

ACE2, angiotensin-converting enzyme 2. *Patient reported SARS-CoV-2 infections. **At least 1 reported SARS-CoV-2 infection at V1, V2, V3, BV0, BV1, BV2, or BV3. <30% and ≥30% denote ACE2 neutralization at V1. N denotes the number of individuals per group.

## Discussion

SARS-CoV-2 has resulted in millions of fatalities worldwide and significant morbidity (11). Both Moderna (mRNA-1273 vaccine) (12) and Pfizer/BioNTech (BNT162b2) (13) vaccines are over 90% effective in preventing severe COVID-19 and elicit high binding and neutralizing antibody titers, as observed in our small cohort of healthy controls. The majority of patients in these studies were healthy volunteers; with only 1% of patients in the Pfizer study having kidney or rheumatologic disease. Patients receiving immunosuppression were excluded from both Pfizer and Moderna studies (12, 13).

Immunocompromised patients with immune-mediated disease are more susceptible to COVID-19 and its complications. There remain many unanswered questions regarding vaccine immunogenicity in this population (14). Will patients mount a similar humoral response to these vaccines under specific conditions? Are vaccines partially effective at preventing symptomatic infection or severe disease in these patients? How long does the humoral response last, and how do various immunosuppressive regimens influence immunogenicity over time? In the present analysis, we longitudinally characterized humoral and, in a subset of patients, cellular immunity following SARS-CoV-2 vaccination. Overall, we found that anti-CD20 antibody therapy was associated with impaired humoral immunity, but T cell responses appeared qualitatively preserved despite immunosuppressant exposure. Time from anti-CD20 therapy to vaccination appeared to be an important risk factor for impaired humoral response, a finding consistent with prior literature (15).

Prior studies have demonstrated that renal dysfunction is associated with impaired germinal center response to T-cell dependent antigens, leading to impaired humoral responses to vaccination (16). Studies have shown retained cellular response following anti-CD20 therapy. For example, while reduced spike-specific and RBD-specific antibody production was observed in 20 patients with multiple sclerosis treated with anti-CD20 monotherapy, the majority of these patients retained antigen-specific CD4+ and CD8+ T-cell responses (17). Time from anti-CD20 infusion and the degree of B-cell reconstitution were associated with the humoral response. Of note, cellular responses, though present, demonstrated some alterations compared to healthy patients, with reductions in T follicular helper cell responses and augmented CD8+ T cell induction being observed (17). In a study of 10 patients with ANCA vasculitis or undifferentiated autoimmune disease, treatment with anti-CD20 therapy was associated with impaired humoral responses but intact T cell responses following SARS-CoV-2 vaccination (18). The majority (90%) of subjects reported only mild COVID-19 infection after a third dose of SARS-CoV-2 vaccine, highlighting the clinical relevance of retained cellular responses in the face of B cell depletive therapy.

In immunosuppressed/immunodeficient patients, two-dose mRNA initial COVID vaccine series may have suboptimal protection against COVID-19. A randomized controlled trial among immunosuppressed/solid organ transplant recipients showed that a third dose of mRNA vaccine provides better immunogenicity than a 2-dose vaccine regimen (19, 20). In August 2021, the FDA amended their Emergency Use Authorization for Pfizer and Moderna COVID-19 vaccines for an additional/booster dose (3^rd^ dose) for improved immunity against COVID-19 (21). Subjects enrolled in this study were immunocompromised/immunodeficient and were eligible for a booster vaccine dose against COVID-19. Our study was not able to investigate the added benefit of booster vaccination due to limited sampling post booster vaccination.

Our study did not demonstrate a statistically significant association between T-ELISpot count and percent ACE2 neutralization, highlighting the possible decoupling of humoral and cell mediated responses in immunosuppressed patients (22). Our finding of retained cellular responses among anti-CD20 treated patients has been reported in multiple other patient populations (23–25). This observation has important implications for the care of immunosuppressed patients, suggesting they may derive some protective benefit from infection or novel variants when vaccinated under immunosuppressed conditions, even those targeting humoral immunity. For example, these T cell responses may have conferred protection to novel coronavirus strains and variants of concern during the pandemic, as has been observed with cross reactivity following infection from seasonal coronavirus strains (26).

Our study has several limitations. First, we include a diverse array of patients with immune-mediated disease, comprising a variety of immunosuppressive agents with varying degrees of humoral and cell-mediated immune impairment. Patients were additionally at different stages in their respective disease courses, with disparate levels of disease activity at time of vaccination and at follow-up visits. Heterogeneity in these factors impacts the overall external validity of our results to specific populations. Additionally, our study was underpowered to observe disease specific associations with vaccine immunogenicity, though such relationships are likely present given known immune dysregulation in many of these conditions. Limited size of the sub-cohort who underwent T cell analysis did limit findings in this group. Quantifying SARS-CoV-2-specific T cell responses and comparing findings across studies is a recognized limitation of these assays due to variability in assay selection and methodology (24, 27, 28).

Despite these limitations, our study’s strengths include a synthesis of clinical data with multiple assay methodologies to characterize the humoral and cell-mediated responses to SARS-CoV-2 vaccination in a vulnerable patient population. An additional strength included enrollment of healthy control subjects allowing for a descriptive comparison. These healthy subjects exhibited responses expected from prior published data in the general population, affirming the utility of our assays and were utilized to establish thresholds of response for the cell-mediated response assays. In summary, vaccination against SARS-CoV-2 during treatment with anti-CD20 antibody therapy was associated with impaired humoral immunity, but T cell responses were qualitatively preserved despite immunosuppressant exposure. Time from anti-CD20 therapy to vaccination appeared to be an important risk factor for impaired humoral response. To optimize vaccine response in patients with immune-mediated disease clinicians should consider the timing of B-cell depletive therapies and COVID vaccination.

## Data Availability

The raw data supporting the conclusions of this article will be made available by the authors, without undue reservation.

## References

[1] Moss P . The T cell immune response against SARS-CoV-2. Nat Immunol. (2022) 23:186–93. doi: 10.1038/s41590-021-01122-w. PMID: 35105982

[2] Glenn DA Hegde A Kotzen E Walter EB Kshirsagar AV Falk R . Systematic review of safety and efficacy of COVID-19 vaccines in patients with kidney disease. Kidney Int Rep. (2021) 6:1407–10. doi: 10.1016/j.ekir.2021.02.011. PMID: 33585728 PMC7870446

[3] Parker EPK Desai S Marti M Nohynek H Kaslow DC Kochhar S . Response to additional COVID-19 vaccine doses in people who are immunocompromised: a rapid review. Lancet Glob Health. (2022) 10:e326–8. doi: 10.1016/S2214-109X(21)00593-3. PMID: 35180408 PMC8846615

[4] Pollard AJ Bijker EM . A guide to vaccinology: from basic principles to new developments. Nat Rev Immunol. (2021) 21:83–100. doi: 10.1038/s41577-020-00479-7. PMID: 33353987 PMC7754704

[5] Deepak P Kim W Paley MA Yang M Carvidi AB Demissie EG . Effect of immunosuppression on the immunogenicity of mRNA vaccines to SARS-CoV-2: a prospective cohort study. Ann Intern Med. (2021) 174:1572–85. doi: 10.7326/M21-1757. PMID: 34461029 PMC8407518

[6] Bange EM Han NA Wileyto P Kim JY Gouma S Robinson J . CD8+ T cells contribute to survival in patients with COVID-19 and hematologic cancer. Nat Med. (2021) 27:1280–9. doi: 10.1038/s41591-021-01386-7. PMID: 34017137 PMC8291091

[7] Narowski TM Raphel K Adams LE Huang J Vielot NA Jadi R . SARS-CoV-2 mRNA vaccine induces robust specific and cross-reactive IgG and unequal neutralizing antibodies in naive and previously infected people. Cell Rep. (2022) 38:110336. doi: 10.1016/j.celrep.2022.110336. PMID: 35090596 PMC8769879

[8] Nanjing GenScript Diagnostics Technology Co. Ltd . Nanjing GenScript Diagnostics Technology Co. Ltd. E-mail: diagnostics@genscript.com Version CE.1.0 Update: 2022.02.17 cPass SARS-CoV-2 Neutralization Antibody Detection Kit [Internet. GenScript. (2022) pp. 1–36. Available online at: https://www.genscript.com/location?href=/gsfiles/techfiles/GS-SOP-CPTS001G-05_L00847-C.pdf?=2022.

[9] Tan CW Chia WN Qin X Liu P Chen MIC Tiu C . A SARS-CoV-2 surrogate virus neutralization test based on antibody-mediated blockage of ACE2–spike protein–protein interaction. Nat Biotechnol. (2020) 38:1073–8. doi: 10.1038/s41587-020-0631-z. PMID: 32704169

[10] Kenward MG Roger JH . An improved approximation to the precision of fixed effects from restricted maximum likelihood. Comput Stat Data Anal. (2009) 53:2583–95. doi: 10.1016/j.csda.2008.12.013. PMID: 38826717

[11] Wang H Paulson KR Pease SA Watson S Comfort H Zheng P . Estimating excess mortality due to the COVID-19 pandemic: a systematic analysis of COVID-19-related mortality, 2020–21. Lancet. (2022) 399:1513–36. doi: 10.1016/S0140-6736(21)02796-3. PMID: 35279232 PMC8912932

[12] Baden LR El Sahly HM Essink B Kotloff K Frey S Novak R . Efficacy and safety of the mRNA-1273 SARS-CoV-2 vaccine. N Engl J Med. (2020) 384:403–16. doi: 10.1056/NEJMoa2035389. PMID: 33378609 PMC7787219

[13] Polack FP Thomas SJ Kitchin N Absalon J Gurtman A Lockhart S . Safety and efficacy of the BNT162b2 mRNA Covid-19 vaccine. N Engl J Med. (2020) 383:2603–15. doi: 10.1056/NEJMoa2034577. PMID: 33301246 PMC7745181

[14] Gragnani L Visentini M Lorini S La Gualana F Santini SA Cacciapaglia F . COVID-19 vaccine immunogenicity in 16 patients with autoimmune systemic diseases. Lack of both humoral and cellular response to booster dose and ongoing disease modifying therapies. J Transl Autoimmun. (2022) 5:100164. doi: 10.1016/j.jtauto.2022.100164. PMID: 36120415 PMC9472465

[15] Troldborg A Thomsen MK Bartels LE Andersen JB Vils SR Mistegaard CE . Time since rituximab treatment is essential for developing a humoral response to COVID-19 mRNA vaccines in patients with rheumatic diseases. J Rheumatol. (2022) 49:644–9. doi: 10.3899/jrheum.211152. PMID: 35232803

[16] Peroumal D Jawale CV Choi W Rahimi H Antos D Li D . The survival of B cells is compromised in kidney disease. Nat Commun. (2024) 15:10842. doi: 10.1038/s41467-024-55187-w. PMID: 39738044 PMC11685736

[17] Apostolidis SA Kakara M Painter MM Goel RR Mathew D Lenzi K . Cellular and humoral immune responses following SARS-CoV-2 mRNA vaccination in patients with multiple sclerosis on anti-CD20 therapy. Nat Med. (2021) 27:1990–2001. doi: 10.1038/s41591-021-01507-2. PMID: 34522051 PMC8604727

[18] Egri N Calderón H Martinez R Vazquez M Gómez-Caverzaschi V Pascal M . Cellular and humoral responses after second and third SARS-CoV-2 vaccinations in patients with autoimmune diseases treated with rituximab: specific T cell immunity remains longer and plays a protective role against SARS-CoV-2 reinfections. Front Immunol. (2023) 14:1146841. doi: 10.3389/fimmu.2023.1146841. PMID: 37180097 PMC10174323

[19] Connolly CM Boyarsky BJ Ruddy JA Werbel WA Christopher-Stine L Garonzik-Wang JM . Absence of humoral response after two-dose SARS-CoV-2 messenger RNA vaccination in patients with rheumatic and musculoskeletal diseases: a case series. Ann Intern Med. (2021) 174:1332–4. doi: 10.7326/M21-1451. PMID: 34029488 PMC8252828

[20] Boyarsky BJ Werbel WA Avery RK Tobian AAR Massie AB Segev DL . Antibody response to 2-dose SARS-CoV-2 mRNA vaccine series in solid organ transplant recipients. JAMA. (2021) 325:2204–6. doi: 10.1001/jama.2021.7489. PMID: 33950155 PMC8100911

[21] U.S. Food and Drug Administation . Coronavirus (COVID-19) update: FDA authorizes additional vaccine dose for certain immunocompromised individuals (2021). Available online at: https://www.fda.gov/news-events/press-announcements/coronavirus-covid-19-update-fda-authorizes-additional-vaccine-dose-certain-immunocompromised (Accessed May 01, 2025).

[22] Mohanraj D Baldwin S Singh S Gordon A Whitelegg A . Cellular and humoral responses to SARS-CoV-2 vaccination in immunosuppressed patients. Cell Immunol. (2022) 373:104501. doi: 10.1016/j.cellimm.2022.104501. PMID: 35299038 PMC8920407

[23] Jyssum I Kared H Tran TT Tveter AT Provan SA Sexton J . Humoral and cellular immune responses to two and three doses of SARS-CoV-2 vaccines in rituximab-treated patients with rheumatoid arthritis: a prospective, cohort study. Lancet Rheumatol. (2022) 4:e177–87. doi: 10.1016/S2665-9913(21)00394-5. PMID: 34977602 PMC8700278

[24] Mrak D Tobudic S Koblischke M Graninger M Radner H Sieghart D . SARS-CoV-2 vaccination in rituximab-treated patients: B cells promote humoral immune responses in the presence of T-cell-mediated immunity. Ann Rheum Dis. (2021) 80:1345–50. doi: 10.1136/annrheumdis-2021-220781. PMID: 34285048

[25] Firinu D Fenu G Sanna G Costanzo GA Perra A Campagna M . Evaluation of humoral and cellular response to third dose of BNT162b2 mRNA COVID-19 vaccine in patients treated with B-cell depleting therapy. J Autoimmun. (2022) 131:102848. doi: 10.1016/j.jaut.2022.102848. PMID: 35714496 PMC9189114

[26] Tarke A Coelho CH Zhang Z Dan JM Yu ED Methot N . SARS-CoV-2 vaccination induces immunological T cell memory able to cross-recognize variants from Alpha to Omicron. Cell. (2022) 185:847–859.e11. doi: 10.1016/j.cell.2022.01.015. PMID: 35139340 PMC8784649

[27] Binayke A Zaheer A Vishwakarma S Singh S Sharma P Chandwaskar R . A quest for universal anti-SARS-CoV-2 T cell assay: systematic review, meta-analysis, and experimental validation. NPJ Vaccines. (2024) 9:3. doi: 10.1038/s41541-023-00794-9. PMID: 38167915 PMC10762233

[28] Gadani SP Reyes-Mantilla M Jank L Harris S Douglas M Smith MD . Discordant humoral and T cell immune responses to SARS-CoV-2 vaccination in people with multiple sclerosis on anti-CD20 therapy. EBioMedicine. (2021) 73:103636. doi: 10.1016/j.ebiom.2021.103636. PMID: 34666226 PMC8520057

