# Supplementary material for: Humoral and cell mediated immune response to SARS-CoV-2 vaccination in patients with immune-mediated diseases
Source: Front Immunol. 2026 May 18;17:1813924. doi: 10.3389/fimmu.2026.1813924 (PMC13223167; doi:10.3389/fimmu.2026.1813924)
Supplement: Supplementary file 1 [file DataSheet1.pdf]

Supplementary Material

Table S1: Summary of statistical measures of % ACE2 neutralization by study visit

Group	Visit Groups	Variable	N	Maximum	Minimum	Median	Lower Quartile	Upper Quartile
**Immune-Mediated Disease Cohort**								
	V0	mean % Neutralization	31	93.17	0	0	0	3.11
	V1	mean % Neutralization	78	97.82	0	46.88	0.645	95.59
	V2	mean % Neutralization	26	97.16	0	10.765	0	89.81
	V3	mean % Neutralization	4	95.57	26.6	71.735	41.87	90.95
	BV0	mean % Neutralization	4	96.28	0	61.775	14.795	95.12
	BV1	mean % Neutralization	33	97.47	0	88.15	19.28	97.18
	BV2	mean % Neutralization	33	97.52	0	96.54	73.52	97.28
	BV3	mean % Neutralization	48	97.3	6.72	95.995	91.28	96.27
**Healthy Control Group**								
	V0	mean % Neutralization	7	16.3	0	4.84	0.36	11.61
	V1	mean % Neutralization	12	97.65	93.16	95.9	94.635	96.5163
	V2	mean % Neutralization	8	95.64	59.77	83.74	76.265	94.085
	V3	mean % Neutralization	1	66.3	66.3	66.3	66.3	66.3
	BV0	mean % Neutralization	4	94.32	60.91	89.705	74.13	93.19
	BV1	mean % Neutralization	3	97.3	97.17	97.26	97.17	97.3
	BV2	mean % Neutralization	3	96.95	95.86	96.07	95.86	96.95
	BV3	mean % Neutralization	4	96.15	96.12	96.135	96.12	96.15

Table S2: Summary of RBD and NTD optical density measurements by time point

Group	Visit Groups	Variable	N	Maximum	Minimum	Median	Lower Quartile	Upper Quartile
**Immune-Mediated Disease Cohort**								
	V0	RBD Optical Density	42	1.9482	0.0726	0.2544	0.1554	0.3207
		NTD Optical Density	42	1.5342	0.0784	0.23005	0.1417	0.3395
	V1	RBD Optical Density	70	2.1309	0.091	0.83595	0.2929	1.8696
		NTD Optical Density	70	1.869	0.0928	0.47475	0.2271	1.4815
	V2	RBD Optical Density	8	1.9241	0.0802	1.589	0.91235	1.84345
		NTD Optical Density	8	1.4576	0.0784	1.09825	0.35435	1.30075
	BV0	RBD Optical Density	1	0.5552	0.5552	0.5552	0.5552	0.5552
		NTD Optical Density	1	0.2004	0.2004	0.2004	0.2004	0.2004
**Healthy Control Group**								
	V0	RBD Optical Density	8	1.8254	0.1117	0.1883	0.155	0.4067
		NTD Optical Density	8	1.5003	0.0741	0.1854	0.11725	0.35015
	V1	RBD Optical Density	12	1.9605	0.1047	1.7637	0.31395	1.88395
		NTD Optical Density	12	1.682	0.1139	1.14735	0.1568	1.5635
	V2	RBD Optical Density	2	1.9653	1.6726	1.81895	1.6726	1.9653
		NTD Optical Density	2	1.4798	0.8709	1.17535	0.8709	1.4798

RBD: receptor-binding domain; NTD: N-terminal domain

Table S3: Demographic and clinical characteristics of rituximab exposed (n=24) or B-cell depleted patients (n=2) at time of vaccination

	% ACE2 Neutralization at V1	
	<30% (N=20)	≥30% (N=6)
**Age,** median (IQR)	64.03(45.84, 69.61)	63.39(46.53, 70.85)
**BMI,** median (IQR)	30.39(24.34, 36.56)	29.63(24.80, 31.00)
**Sex,** female	17(85%)	3(50%)
**Race**		
White	20(100%)	5(83.33%)
Multiracial/Other	0(0.00%)	1(16.67%)
**Ethnicity**		
Not Hispanic/Latino	19(95%)	5(83.33%)
Hispanic/Latino	1(5%)	1(16.67%)
**Disease types**		
Dermatological Disease	4(20%)	2(33.33%)
Gastrointestinal Disease	4(20%)	3(50%)
Vasculitis	12(60%)	1(16.67%)
**Comorbidities**		
Hypertension	7(35%)	5(83.33%)
DM	0(0.00%)	1(16.67%)
Liver cirrhosis	0(0.00%)	0(0.00%)
Hepatitis A/B/C	0(0.00%)	0(0.00%)
Cancer	2(10%)	0(0.00%)
Tobacco Exposure	1(5%)	1(16.67%)
Other	1(5%)	0(0.00%)
**Vaccine type**		
Pfizer	15(75%)	2(33.33%)
Moderna	4(20%)	4(66.67%)
Johnson & Johnson	1(5%)	0(0.00%)
**Prior COVID19 infection before vaccination**	0(0.00%)	0(0.00%)
**Disease statuses**		
Active disease	6(30%)	4(66.67%)
Remission	14(70%)	2(33.33%)
**Time from Rituximab infusion to First SARS-CoV-2 Vaccine* (months),** median (IQR)	2.94(2.11, 4.62)	4.13(2.63, 6.45)

ACE2: angiotensin-converting enzyme 2

*One patient was vaccinated 2 days prior to a rituximab infusion and assigned a value of -2 days.

Table S4: Univariate and multivariable logistic regression model for odds of percent ACE2 neutralization ≥30% at timepoint V1.

	**Univariate Analysis**		**Multivariable Model** (N=75)	
	**OR (95%CI)**	**P value**	**OR (95%CI)**	**P value**
**Age**	1.00(0.98, 1.02)	0.9636	1.00(0.97, 1.03)	0.8981
**Sex**				
Male	ref		ref	
Female	0.36(0.12, 1.07)	0.065	0.30(0.07, 1.29)	0.1044
**BMI**	1.00(0.94, 1.07)	0.963	1.03(0.95, 1.12)	0.5060
**Race**				
White	ref		ref	
Black	2.18(0.52, 9.25)	0.2894	1.47(0.25, 8.69)	0.6714
Other	3.74(0.40, 35.47)	0.2502	5.79(0.46, 73.16)	0.1749
**Initial vaccine type**				
Pfizer	ref		ref	
Moderna	1.65(0.64, 4.25)	0.2977	1.91(0.58, 6.25)	0.2856
**Immunosuppression**				
No Immunosuppression	ref		ref	
Rituximab	**0.13(0.03, 0.53)**	**0.0042**	**0.15(0.03, 0.71)**	**0.0166**
MMF/Azathioprine	0.73(0.19, 2.83)	0.6484	0.74(0.14, 3.79)	0.7175
Other*	4.17(0.42, 41.78)	0.2250	4.73(0.37, 60.05)	0.2305
**Comorbidity Count**	0.94(0.51, 1.73)	0.8318	0.73(0.31, 1.74)	0.4815
**Disease Type**				
Vasculitis	ref		--	
Dermatologic	0.65(0.15, 2.79)	0.5603	--	
GI	0.81(0.05, 13.92)	0.8842	--	
Glomerular Disease	0.61(0.18, 2.09)	0.429	--	
Rheumatologic	4.05(0.78, 21.02)	0.0962	--	
**Disease Status at Time of Vaccination (missing 4)**				
Active	ref		--	
Remission	0.52(0.18, 1.51)	0.2281	--	
**Labs at V0**			--	
eGFR (missing 25)	1.01(0.99, 1.04)	0.3772	--	
UPCR (missing 57)	0.50(0.16, 1.56)	0.2309	--	
Serum albumin (missing 29)	0.57(0.10, 3.27)	0.5238	--	

ACE2: angiotensin-converting enzyme 2

Table S5: Mixed effects model of longitudinal % ACE2 neutralization response with interaction of immunosuppression category and time.

Effect		Estimate	SE	P
**Intercept**		24.4	15.9	0.1272
**Month**		23.2	7.1	0.0015
**Month*Month**		-1.8	0.9	0.0373
**Immunosuppression**	Rituximab	3.0	13.9	0.831
	MMF/Azathioprine	24.9	16.2	0.1299
	Other Immunosuppression	-11.7	24.8	0.6402
	No Immunosuppression	ref	.	.
**Months* Immunosuppression**	Rituximab	-23.1	8.0	0.0047
	MMF/Azathioprine	-16.2	8.5	0.0594
	Other Immunosuppression	13.5	13.2	0.3098
	No Immunosuppression	ref	.	.
**Month*Month* Immunosuppression**	Rituximab	1.9	0.9	0.0399
	MMF/Azathioprine	1.4	0.9	0.1508
	Other Immunosuppression	-2.0	1.6	0.2184
	No Immunosuppression	ref	.	.
**Age (yr)**		-0.2	0.2	0.3796
**Sex**	Female	-9.3	8.1	0.2561
	Male	ref	.	.

ACE2: angiotensin-converting enzyme 2; MMF: mycophenolate mofetil

Table S6: Summary of ELISpot assay

Group	Visit	Variable	N	N Miss	Median	Lower Quartile	Upper Quartile	Minimum	Maximum
**Immune-Mediated Disease Cohort**									
	**V0**	**CEFX**	4	0	231.5	156.25	366	81.5	500
		**RBD**	4	0	2.25	2	3.75	2	5
	**V1**	**CEFX**	28	1	149.5	73.25	291	14.5	500
		**RBD**	29	0	16	12	37	4	179
	**V2**	**CEFX**	12	0	268.75	114.25	457.75	33.5	500
		**RBD**	12	0	17.5	9.25	28	3.5	116.5
	**BV0**	**CEFX**	2	0	337.75	257	418.5	257	418.5
		**RBD**	2	0	12	6	18	6	18
	**BV1**	**CEFX**	10	0	346	109.5	500	20.5	504
		**RBD**	10	0	25.5	12	45	4	76.5
	**.BV2**	**CEFX**	7	0	352.5	21.5	500	18	500
		**RBD**	7	0	16.5	5.5	39	1	72
	**BV3**	**CEFX**	1	0	26	26	26	26	26
		**RBD**	1	0	6.5	6.5	6.5	6.5	6.5
**Healthy Control Group**									
	**V0**	**CEFX**	5	0	231	143	232	42	331.5
		**RBD**	5	0	2.5	2.5	3	1	5
	**V1**	**CEFX**	7	0	167.5	75.5	291	33.5	337
		**RBD**	7	0	16	4.5	23	3	23.5
	**V2**	**CEFX**	3	0	195	155	297.5	155	297.5
		**RBD**	3	0	10.5	3.5	18.5	3.5	18.5
	**BV0**	**CEFX**	1	0	257	257	257	257	257
		**RBD**	1	0	18	18	18	18	18
	**BV1**	**CEFX**	1	0	296.5	296.5	296.5	296.5	296.5
		**RBD**	1	0	23.5	23.5	23.5	23.5	23.5

Figure S1: Percent ACE2 neutralization by study visit

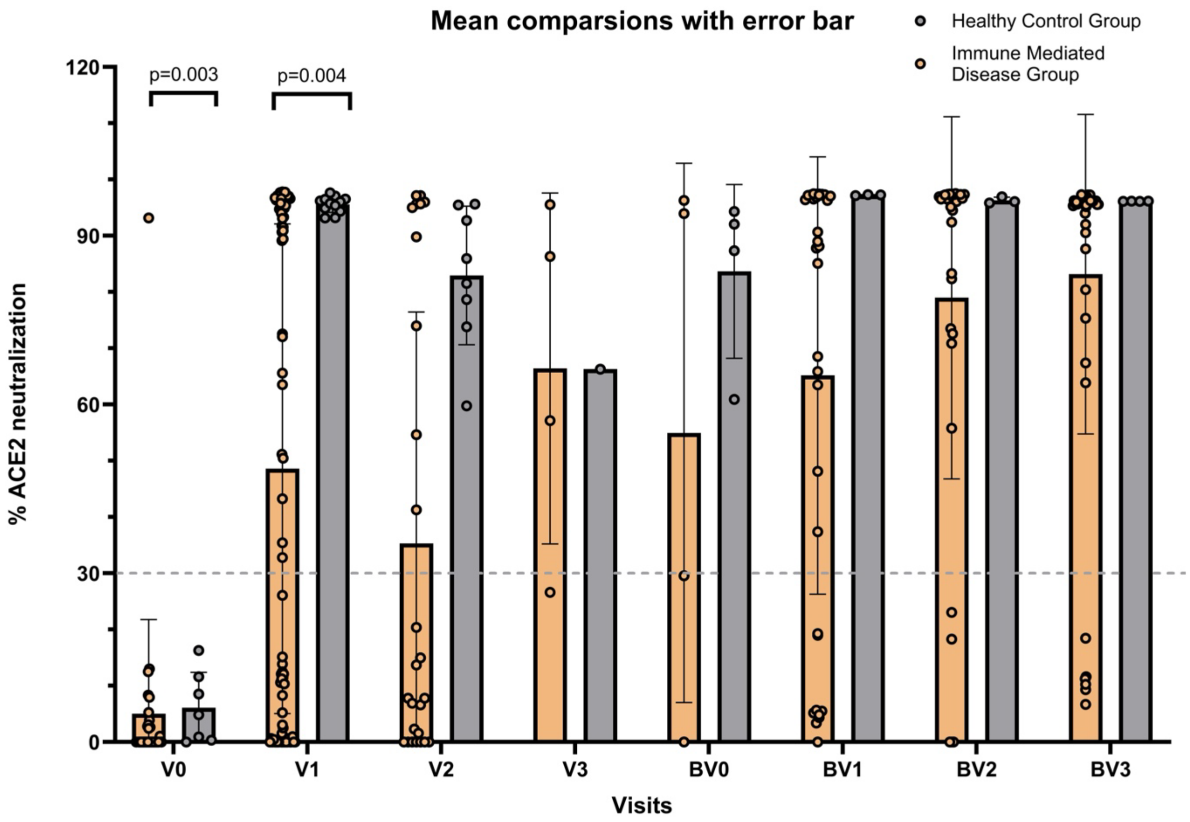

ACE2: angiotensin-converting enzyme 2

Figure S2: Model-estimated ACE2 neutralization trajectories by immunosuppression group

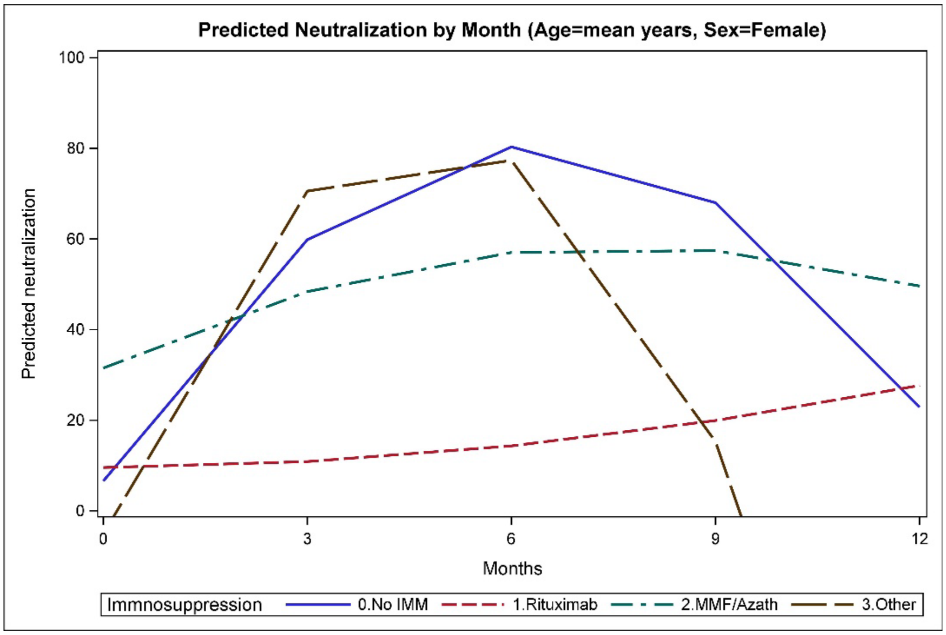

IMM: immunosuppression
